# Analysis of Vocal Fold Function From Acoustic Data Simultaneously Recorded With High-Speed Endoscopy

**DOI:** 10.1016/j.jvoice.2012.02.001

**Published:** 2012-11

**Authors:** Michael Döllinger, Melda Kunduk, Manfred Kaltenbacher, Sabine Vondenhoff, Anke Ziethe, Ulrich Eysholdt, Christopher Bohr

**Affiliations:** ∗ Department for Phoniatrics and Pedaudiology, University Hospital Erlangen, Erlangen, Germany; † Department for Communications Sciences and Disorders, Louisiana State University, Baton Rouge, Louisiana; ‡ Applied Mechatronics, Institute of Smart System-Technologies, Alps-Adriatic University Klagenfurt, Klagenfurt, Austria; § ENT-Hospital Erlangen, University Hospital Erlangen, Erlangen, Germany

**Keywords:** Acoustic analysis, Endoscopic high-speed imaging, Vocal fold function

## Abstract

**Summary:**

Acoustic and endoscopic voice assessments are routinely performed to determine the vocal fold vibratory function as part of the voice assessment protocol in clinics. More often than not these data are separately recorded, resulting in information being obtained from two different phonation segments and an increase of time for the voice evaluation process. This study explores the use of acoustic data, simultaneously recorded during high-speed endoscopy (HSE), for the evaluation of vocal fold function.

**Patients and Methods:**

HSE and acoustic data were recorded from the subjects simultaneously during sustained phonation. The data included voices of 73 healthy subjects, 148 paresis, 210 functional dysphonias, and 119 benign lesions of vocal folds. For this study, only acoustic data were analyzed using *Dr. Speech* software (Tiger electronics Inc., MA). Twelve parameters were computed; 82% of the acoustic voice recordings could be analyzed. Statistical analysis was performed with *SPSS* 17.0.

**Results:**

Acoustic data was easily recorded simultaneously allowing analyses of the same phonation segment to determine vocal fold function and therefore eliminating the need for another voice recording. The acoustic voice parameters differed between genders in the healthy voice group. Most of the parameters showed significant differences between healthy and pathological groups.

**Conclusion:**

Simultaneously recorded endoscopic and acoustic data is valuable. Differentiation between healthy and pathological groups was possible using acoustic data only. We suggest that the synchronously recorded acoustic signal is of sufficient quality for objective analysis yielding reduced examination time.

## Introduction

Evaluation of voice quality in daily clinical practice is a complex process. There is an increasing need for objective and comparable techniques to quantify voice impairments in dysphonic patients. The clinical voice evaluation protocols may include acoustic, aerodynamic, endoscopic, and perceptual (by trained listener, eg, CAPE-V^1^) and patient-self assessment (eg, Voice Handicapped Index^2^) techniques.

The objective evaluation of the acoustical signal obtained from patients is an important aspect of clinical voice evaluation protocols. *Jitter* (frequency perturbation), *Shimmer* (amplitude perturbation), and *HNR* (harmonic-to-noise ratio) are frequently used perturbation measures.^3–5^ These methods are traditionally based on linear mathematical principles and are thought to be limited to nearly periodic signals and might be inappropriate for aperiodic voice signals.^6^ Hence, studies have examined nonlinear dynamic analysis to investigate the behavior of the human voice.^7^ Methods such as correlation dimension, phase space reconstruction, or Lyapunov exponents have shown potential applicability in clinical voice analysis, as they are capable to analyze irregular and chaotic oscillations.^8,9^ Jiang et al^10^ emphasized that perturbation measures and nonlinear dynamic analysis provide complementary information. A combination of these two methods can provide more precise results especially in analysis and description of patients with severe voice disorders.

Recently, high-speed digital endoscopy (HSE) has been made available for clinical evaluation as part of clinical voice assessment protocol.^11^ HSE overcomes several disadvantages of stroboscopy and enables the clinician to record the oscillating vocal folds in real time during phonation.^11^ HSE is particularly useful to visualize and to quantify pathologies that affect the dynamic behavior of the vocal folds. HSE is also applicable for highly disturbed and aperodic signals.^12^ The main disadvantages of HSE are the high acquisition costs and a lower image quality than Stroboscopy.^13^ Up till now, there are no standardized and widely accepted techniques or parameters for an objective evaluation of HSE videos.^14^ However, several research groups have already initiated the objective evaluation of HSE data.^11,13,14^

Future studies combining simultaneous recording of HSE and the acoustical signal would be desirable. This type of voice data collection and analysis could lead to a more robust evaluation of vocal pathologies and may yield improvements in clinical voice protocols for the assessment and diagnosis of voice conditions. In addition, this approach will further shorten the time for voice data collection for assessment and allow clinicians to have a direct connection between the acoustic voice signal and the vocal fold vibrations. As a first step, we analyzed only the acoustic recordings of normal and pathological voices, which were simultaneously acquired during HSE recordings of vocal folds during sustained phonation. We applied traditional objective voice assessment with the commercial analysis software *Dr. Speech* (Tiger electronics Inc., WA).^15^ For the quantitative description of the voice signal, several widely used acoustic parameters including perturbation measures such as *HNR*, *Jitter* (%), and *Shimmer* (%) are used. The goal of the analyses is as follows:

1. To determine the ability of objective acoustic parameters in differentiating between disordered voices with varying causes and healthy voices, when the acoustical signal was synchronously recorded during endoscopy.
2. To identify potential correlations between separate voice measures and to reduce the number of parameters.
3. To determine the ability of a linear discriminate analysis (LDA) in discriminating between healthy voices and pathological voices.
4. To determine the ability of an LDA in discriminating between pathological voices with varying causes.

## Method

### Database

All voice samples were selected from the database from the Department of Phoniatrics and Pediatric Audiology at the University Hospital Erlangen, Germany. After institutional review board’s approval in 2003, they had been collected in clinical routine over the last years during out- and in-patient treatment. Totally 672 voice samples were evaluated, which consisted of 438 women (aged 16–78 years) and 234 men (aged 16–77 years). The recordings were distributed in two main groups.

One group contained 80 (48 women, 32 men) healthy control subjects who had neither history nor clinical signs of voice disorder. The other group included dysphonic patients and was subdivided into patients with organic and nonorganic voice disorders according to the diagnosis made by a laryngologist. The group of organic voice disorders contained 338 subjects (202 women, 136 men) of patients with paralysis of the recurrent laryngeal nerve (RLNP) and with anatomical changes (AC) of the vocal fold such as polyps, Reinke’s edema, cysts, nodules, and granulomas. The last group included 254 voice samples (188 women, 66 men) with functional dysphonia (FD). In this group, no morphological changes of the vocal folds could be identified but the voices were still perceptually hoarse.

### Recording technique and data processing

Acoustic data was obtained *via* a condenser microphone (Brül & Kajaer 4129; Brül & Kajaer, Copenhagen, Denmark), which was mounted on a rigid 90° laryngoscope together with a high-speed camera (HS Endocam; Richard Wolf GmbH, Knittlingen, Germany). The distance between microphone and patients mouth was 20 cm. Hence, it was possible to acquire acoustic data simultaneously to high-speed imaging of the vocal folds. During the voice evaluation, the patients were asked to perform sustained phonation of the German vowel /a/ at their comfortable pitch and loudness levels.

From the simultaneous recordings of HSE and acoustic data, only the audio data was analyzed for this study. The 672 audio files were inspected with the software *Audacity*^16^ and a sequence of sustained phonation of minimum length of ≥300 milliseconds and maximum length of 1.84 seconds was extracted. The new wav-files were stored under 16 bit and ready to be analyzed with *Dr. Speech* analyzing software.^15^

### Analyzed parameters

In recent literature, a large number of objective acoustic parameters have been introduced. However, it is still not clear which combination of these measures is particularly suitable for a characterization of disordered voices. To determine the ability of objective acoustic parameters in characterizing and differentiating disordered and healthy voices, we calculated objective voice quality measures by *Dr. Speech* commercial voice function assessment software. From the 16 output parameters, the 12 numerical parameters were chosen for further analysis. The chosen measures are widely used as measures for voice quality and voice function. They contain two frequency perturbation measures (*Jitter* [%], *Shimmer* [%]), three noise parameters (*HNR* “*harmonic-to-noise ratio*,” *SNR* “*signal-to-noise ratio*,” and *NNE* “*normalized noise error*”), and seven frequency parameters (*STD F_0_*, *Mode F_0_*, *STD period*, *Mean amplitude*, *STD amplitude*, *F_0_ tremor*, and *Amplitude tremor*).

### Statistics

Statistical analysis was performed using *SPSS* statistics 17.0 (IBM Deutschland GmbH, Ehningen, Germany). For descriptive and comparative analysis, we divided the 550 data sets that were appropriate for analysis into four groups. All statistics are applied to these four separated groups, Table 1:

1. Healthy subjects with normal voices (*Controls*).
2. *RLNP*, which contains all data sets from right and left RLNP and on both sides.
3. FD.
4. AC of the vocal fold which contains the data sets from polyps, Reinke’s edema, cysts, nodules, and granulomas.

As the 12 objective measures are on a numerical scale, the mean values and the standard deviations for control subjects and pathological groups were computed.

To check the distribution of the parameters, Komogorov-Smirnov tests were applied. In case of a non-Gaussian distribution, the nonparametric Mann-Whitney *U* test was performed. In case of a Gaussian distribution, a Student’s *t* test for independent samples was applied to analyze differences between gender groups and between healthy and pathological groups. *P*-values below 0.05 (∗) were considered as statistically significant and below 0.001 (∗∗) as highly statistically significant.

A factor analysis (FA) test was performed using the 12 parameters obtained by *Dr. Speech* to identify which parameters contain similar information (ie, parameters loading within the same factor) and to analyze if there is any variance within the parameters for the data.^17^

Finally, a linear discriminant analysis (LDA)^17^ was applied for each gender group separately to discriminate between healthy subjects and the pathological group. As the group size has to be equal for LDA, we randomly chose all 43 female *Controls* and randomly 43 within RLNP, FD, and AC (total: 172 patients). For males, we chose all 30 *Controls* and randomly 30 within the three other groups (total: 120 subjects).

## Results

In the course of the acoustic evaluation with *Dr. Speech*, 122 (18%) of the 672 data sets had to be excluded because the extracted sample was too short or in such a high grade disturbed that an accurate analysis was impossible or not all values could be computed. In the end, 550 data sets, composing of 82% of the raw data, including 73 healthy subjects (43 women, 30 men) and 477 with voice disorders (310 women, 167 men) remained for statistical analysis, Table 1.

### Descriptive analysis

Tables 2 and 3 display the results of the descriptive analysis of the 12 applied objective voice quality measures for control subjects (Table 2) and the three pathological groups (Table 3). The mean values and standard deviations are given. The gender separation is done for the entire analysis because of known differences between male and female voice parameters,^18–20^ which is confirmed by our results for the synchronously recorded acoustic signal. The tests show that 11 parameters except *STD amplitude* differ between men and women within the healthy *Controls* (Table 2).

### Statistical comparison between controls and pathological groups

Table 3 also displays statistical differences marked with (∗) and (∗∗) between control subjects and the three pathological groups separated for men and women. Parameter values that did not show statistical differences between controls and the specific pathology are bolded.

### Paralysis of the recurrent laryngeal nerve

For men, all values differ at least significantly between normal and pathological voices. Women show no significant differences for *Mode F_0_*, *F_0_ tremor*, and *Amplitude tremor*. For *Mean amplitude*, the difference is significant, whereas in all other parameters, the difference is highly significant (Table 3).

### Functional dysphonia

Table 3 presents the results for the comparison of FD and healthy voices. For men, there are no significant differences for the average values for *STD period*, *F_0_ tremor*, and *Amplitude tremor*. With the exception of the three highly significant values for *NNE*, *STD F_0_*, and *Mode F_0_*, all other six measures show a significant difference. For women, the comparison of healthy voices and FD show no significant difference for *Mode F_0_*, *F_0_ tremor*, and *Amplitude tremor*, whereas *STD F_0_*, *STD period*, and *STD amplitude* have *P*-values <0.001. The remaining six parameters show significant differences.

### Anatomical changes

Men and women did not show any significant differences in the *F_0_ tremor* parameter. Additionally, women did not show significant differences in *Mean amplitude* and *Amplitude tremor*. All other parameters were significantly different between controls and AC groups for both genders (Table 3).

Table 3 demonstrates that for the analyzed parameters, the seven parameters *Jitter* (%), *Shimmer* (%), *HNR*, *SNR*, *NNE*, *STD F_0_*, and *STD amplitude* are most suitable for differentiating normal and disordered voices. These seven parameters show statistically significant differences between healthy and pathological subjects in all three groups (RLNP, FD, and AC) for men and women. Overall, the measures separate better for men than for women as can be seen in Table 3.

### Factor analysis

The acoustic parameters for all patients (353 female, 197 male) were evaluated by FA, performed separately for men and women. Parameters loading on a specific factor are highlighted in Table 4. Three factors are determined, whereof the first factor is dominant as it explains 51.0% of the total variance for female samples and 56.8% for male samples. Table 4 shows the 12 measures and their factor loadings. For both gender groups, factor 1 combines the two perturbation measures *Jitter* and *Shimmer*, the three noise parameters *HNR*, *SNR*, and *NNE*, and four parameters of absolute frequency *STD F_0_*, *STD period*, *Mean amplitude*, and *STD amplitude*. *HNR*, *SNR*, and *Mean amplitude* show negative factor loadings, whereas the other measures show positive values.

Only one parameter loads on factor 2 for women (*Amplitude tremor*) and for men (*Mode F_0_*). For factor 3, the percentages are 11.2% for women (*mode F_0_*, *F_0_ tremor*) and 10.6% for men (*Amplitude tremor*, *F_0_ tremor*).

### Linear discriminate analysis

The applied LDA investigated how useful a combination of specific measures is in classifying (ie, identifying) normal and pathological voices for women and men separately. As not all 12 variables excluded high correlations (<0.7) as tested with Kendall’s Tau correlation coefficient, two of them were removed for LDA analysis because of unnecessary redundancy: *SNR* and *STD amplitude*.

All LDA classification was performed with each group containing the same amount of women (42) and men (30). These numbers were chosen based on the minimum number of subjects in male/female groups. Based on the parameter assignment by the FA, we divided the LDA into three cases using different sets of acoustic parameters:

**Case 1:** LDA with the 10 remaining acoustic measures.

**Case 2:** LDA with eight parameters ignoring *F_0_ tremor* and *Amplitude tremor*. (Parameters within factor 1 and parameter Mode F_0._)

**Case 3:** LDA without the measures *F_0_ tremor*, *Amplitude tremor*, and *Mode F_0_*. (Only parameters within factor 1; total of seven parameters.)

**Figure 1:**
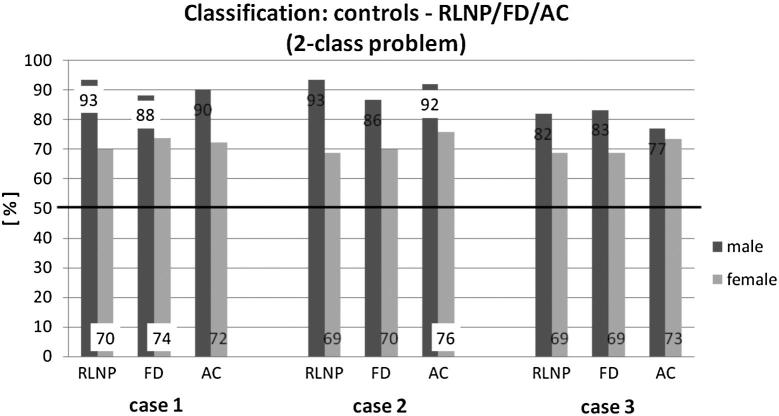
Classification results for linear discriminant analysis for the two-class problems: Controls vs RLNP, Controls vs FD, and Controls vs AC. Cases 1–3 refer to the three cases of different parameter grouping mentioned in the text. The data labels are the percentages of correct classified subjects. The best classification result for each pathological group is marked white. The black line illustrates the baseline accuracy.

First, for each of the three cases, we constructed two-class problems: Figure 1 shows the LDA results where the controls were compared with each individual pathology. The horizontal line at 50% shows the classification correctness for random classification.

As Figure 1 shows, the classification results for women (69–76%) are lower than for men (77–93%). Case 1 and Case 2 show similar good classification accuracy values. Case 3 does show decreased classification accuracy. Males exhibit better LDA results than females. The following is a brief description of the results of the LDA separated for each of the three cases (ie, different parameter sets):

**Case 1:** This case shows for RLNP (males) and FD (females), the highest percentages of correctly grouped data sets for both gender groups. The worst classification result was for the female group of RLNP (70%).

**Case 2:** RLNP men and AC women have the highest percentage in this case. The worst classification result was again for the female group of RLNP (69%).

**Case 3:** The worst classification results are achieved.

**Figure 2:**
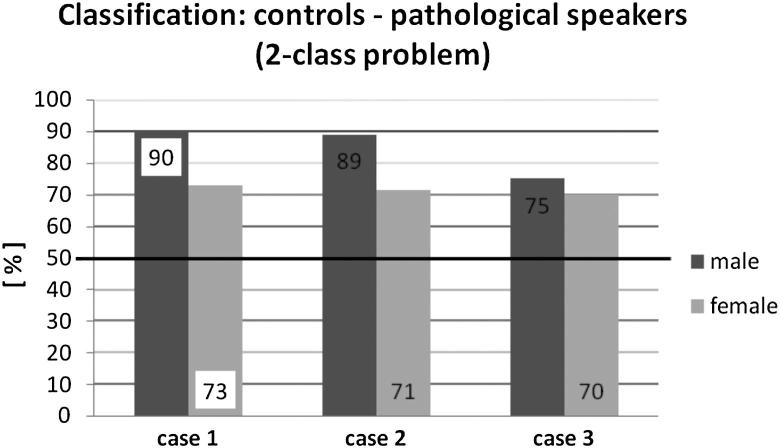
Classification results for the linear discriminant analysis for the two-class problem: Controls vs all pathologies in a group. The numbers 1–3 refer to the three cases of parameter grouping mentioned in the text. The data labels inside the columns are the percentages of correct classified subjects with the best classification result for each pathological group marked white. The black line illustrates the baseline accuracy.

Figure 2 shows the LDA results for comparing the controls with a group containing subjects (equally distributed, randomly chosen) from all three pathological groups. Again, the classification results for males are better than they are for females. Both gender groups have the best classification results for Case 1 (10 parameters). The worst percentage of correct classification is for male and female subjects for Case 3 (only seven parameters).

**Figure 3:**
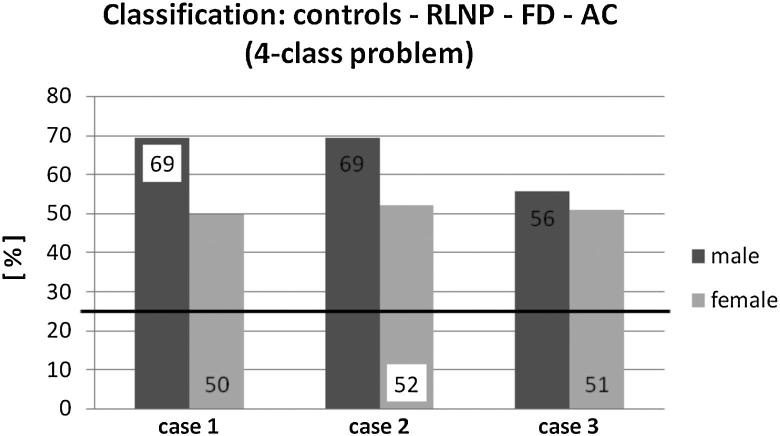
Classification results for linear discriminant analysis for the four-class problem: Controls vs RLNP vs FD vs AC. The numbers 1–3 refer to the three cases of parameter grouping mentioned in the text. The data labels inside the columns are the percentages of correct classified subjects with the best classification result for each pathological group marked white. The black line illustrates the baseline.

Figure 3 shows the LDA classification results for the four-class problem: The *Controls* and the three pathologies (RLNP, FD, and AC) were separated. The lowest classification accuracies were obtained in this group (two groups in Figures 1 and 2, here four different groups). The horizontal line at 25% shows the accuracy for random classification. Again, the LDA results for males are better (56–69%) than for females (50–52%). Case 1 and Case 2 show similar performance, whereas Case 3 exhibits less performance than the other two.

## Discussion

In this study, we investigated if acoustic data acquired simultaneously to HSE can be analyzed and still differentiate between the normal and pathological voices. The computed and compared commonly applied acoustic voice parameters between a healthy control group and three pathological groups demonstrate that presence of the endoscope did not seem to prevent the differentiation of normal and pathological voices from the acoustic signal. In addition, we demonstrated that:

1. Perturbation measures could be computed in 82% of all clinical data applying commercially available signal analysis software (*Dr. Speech*). Performing enhanced signal processing (eg, improved peek-picking algorithms) may even increase this performance value in future.
2. Comparing healthy females versus healthy males, statistically significant differences in 11 out of 12 parameters are present, Table 2. Most of the parameters showed differences between healthy and pathological groups, Table 3. Pathologies could be better differentiated for men than for women, Table 3 and Figures 1–3.
3. LDA classification showed, good differentiation between healthy and pathological groups (Figures 1 and 2), but the differentiation between different voice pathology groups was limited, Figure 3.

A small number of data sets did not provide acoustic values (18%); this is likely because of the extracted sample being too short in duration, or of such low quality that accurate analysis was not possible, or not all values could be computed. This substantiates Inwald et al,^21^ who evaluated 496 clinical HSE videos. By perceptual analysis of the HSE recordings, they found that healthy and dysphonic voices of FD and RLNP groups showed acceptable small nonperiodic vibrations of the vocal folds reflecting the periodicity of the acoustical signal. Those vibrations correspond to *type 1*: nearly periodic and *type 2*: signals with subharmonics and modulations according to Titze’s classification.^6^

In clinical practice, mostly perturbation measures such as *Jitter*, *Shimmer*, *SNR*, and *HNR* are applied as objective measures to assess voice disorders.^22^ For the calculation of these measures, popular commercial voice assessment programs such as the *Multidimensional Voice Program* (*MDVP*)^23^ and *Dr. Speech*^15^ (KayPentax, Montvale, NJ) are applied. In this work, we used 12 common objective voice quality measures, calculated by *Dr. Speech*. The control group showed statistically significant differences between men and women concerning all parameters expect *STD amplitude*. Pützer^18^ also examines voice parameters such as *relative Jitter* (%), *relative Shimmer* (%), and *STD F_0_* for 150 healthy male and 150 female speakers with *MDVP* analyzing software. Our findings are in agreement for values of *relative Jitter* (%), *relative Shimmer* (%), and *STD F_0_*, which were smaller for male subjects. Pützer^18^ and our studies demonstrate highly significant differences between healthy men and women for *STD F_0_* and *Jitter* and significant difference for *Shimmer*.

Smits et al^19^ analyzed voices of 120 healthy subjects with *Computerized Speech Laboratory* (*CSL*; KayPentax, Montvale, NJ) and *Dr. Speech*. They built four age groups from which the first one combines subjects from 20 to 30 years. Comparing our Control group values (females: 19–26 years, males: 16–21 years) with Smits’s group showed that our *Jitter* (%) and *Shimmer* (%) values are lower, whereas our *HNR* (dB) values are higher in this study. These differences can be ascribed to different levels of acoustic noise in the environment^24^ or different recording setups.^25^ Smits et al^19^ recorded all voice samples with an ECM-717 electric condenser microphone and a digital audiotape (Sony DAT recorder, Walkman type, sampling rate of 48 kHz). However, further studies are needed for clarification.

### Differentiation between healthy and pathological groups

All results of the applied LDA are clearly above the baseline accuracy of 50% in a two-class problem and above 25% in a four-class problem. Our results show an accurate classification rate for the comparison of controls with pathological voices of 90% for men and 73% for women in the parameter set Case 1, Table 2. Werth et al^26^ also investigated the diagnostic potential of perturbation measures (*Jitter* [%], *Shimmer* [%], *HNR* [dB], and *NNE* [dB]). They reported an average classification accuracy of 67.5% for men and 63.6% for women. Differences in classification accuracy between our and their studies for both gender groups could be due to fewer number of voice parameters in Werth et al’s study. They only applied four objective voice parameters instead of 10 parameters (Case 1). We experienced the same trend, when reducing the number of parameters (as in the seven parameters used in Case 3): the accuracy of LDA classification decreased. Analyzing female subjects, Voigt et al^27^ performed a two-class LDA with three different decision tasks (Healthy vs Paresis left, Healthy vs paresis right, Healthy vs Pathological) using Phonovibrography to analyze HSE data. Considering much more parameters than our study, Voigt et al^27^ achieved an average classification accuracy of 93%. Our comparison of *Controls* versus RLNP reached the same accuracy for men and a lower percentage for women (70%), Figure 1. To reduce computational time and to keep the analysis descriptive, the future goal will be to find the balance between the number of analyzed parameters and the accuracy level for classification purposes.

In summary, this study further demonstrates that acoustic analyses can differentiate between normal and disordered voices despite the presence of endoscopy during simultaneous recording of HSE and audio signal. In addition, LDA classification method yields higher classification rates for males than females between the normal and disordered voices. This study suggests the simultaneously recording of HSE and acoustics for a combined analysis yielding improved objective diagnostics in future.

### Limitations

The classification was not as successful for differentiating among the varying individual pathologies as for differentiating healthy versus all pathological voices. This may be based on the limited informative value of the acoustic signal regarding separation of pathologies, a too short analyzed time window where certain pathologies do not exhibit their specific characteristics, or on the fact, that some pathologies expose their disordered characteristics only at certain frequencies.^28^ Further studies are needed to determine the exact changes within the acoustic parameters when obtained simultaneously with a rigid or flexible endoscope and without. Studies where both image and acoustic data are considered for analysis^29^ and where classification will have to be performed, which will probably improve the so far achieved results.

## Figures and Tables

**Table 1:** Summary of Number of Subjects and Grouping: RLNP, AC, FD, and Healthy Subjects (Controls)

Phathologies	Female	Male	Total	Missing	Grouping
Paralysis right	50	45	95	20	RLNP
Paralysis left	45	43	88	26
Paralysis on both sides	6	8	14	3

Polyps	37	20	57	12	AC
Reinke’s edema	32	2	34	3
Cysts	7	9	16	3
Nodules	23	0	23	2
Granuloma	2	9	11	2

Functional dysphonia	188	66	254	44	FD

Male controls	0	32	32	2	Controls
Female controls	48	0	48	5

Total	438	234	672	122	

*Note:* The numbers within the column “Missing” represent the subjects, which could not be analyzed by *Dr. Speech* software.

*Abbreviations:* RLNP, paralysis of recurrent laryngeal nerve; FD, functional dysphonia; AC, anatomical changes.

**Table 2:** Mean and STD for the 12 Computed Acoustic Parameters for the *Controls* are Given

Acoustic Values	Controls			
Female		Male	
Mean	STD	Mean	STD
Jitter (%)	0.31∗∗	0.13	0.24	0.25
Shimmer (%)	2.83∗	1.30	2.22	2.31
HNR (dB)	24.32∗	4.25	26.81	5.19
SNR (dB)	24.36∗	4.22	26.85	5.09
NNE (dB)	−9.26∗	4.54	−12.67	4.51
STD F0 (Hz)	2.26∗∗	1.06	1.59	1.34
Mode F0 (Hz)	260.44∗∗	47.55	142.23	34.34
STD period (ms)	0.04∗∗	0.02	0.08	0.04
Mean amplitude (%)	86.92∗	5.28	90.42	4.30
STD amplitude (%)	**5.63**	2.05	5.17	2.82
F0 tremor (Hz)	6.44∗	2.86	4.85	2.21
Amplitude tremor (Hz)	7.30∗∗	3.45	4.17	1.83

*Notes:* The mean values under the female column marked with (∗) show a significant and (∗∗) shows a statistical highly significant difference between both gender groups within the *Controls*. The bold value is the only one that has no significant difference.

*Abbreviations:* STD, standard deviation; Mean, mean values; HNR, harmonic-to-noise ratio; SNR, signal-to-noise ratio; NNE, normalized noise error.

**Table 3:** Mean and STD for the 12 Objective Acoustic Values for Males and Females, Shown for the Three Pathology Groups: RLNP, FD, and AC of the Vocal Fold

Acoustic Values	RLNP			
Female		Male	
Mean	STD	Mean	STD
Jitter (%)	1.07∗∗	1.17	1.41∗∗	1.63
Shimmer (%)	5.96∗∗	4.43	7.08∗∗	5.47
HNR (dB)	18.33∗∗	7.23	16.37∗∗	8.47
SNR (dB)	18.64∗∗	6.79	16.88∗∗	7.89
NNE (dB)	−5.55∗∗	5.62	−4.64∗∗	5.26
STD F0 (Hz)	5.63∗∗	6.54	4.99∗∗	4.90
Mode F0 (Hz)	**240.49**	77.91	185.91∗∗	53.50
STD period (ms)	0.13∗∗	0.23	0.18∗	0.22
Mean amplitude (%)	82.15∗	8.18	80.78∗∗	11.00
STD amplitude (%)	8.12∗∗	3.35	8.51∗∗	4.37
F0 tremor (Hz)	**6.82**	4.01	7.66∗	4.04
Amplitude tremor (Hz)	**7.90**	4.09	6.43∗	3.72

Acoustic Values	FD			
Female		Male	
Mean	STD	Mean	STD
Jitter (%)	0.814∗	1.16	0.48∗	0.59
Shimmer (%)	4.53∗	3.82	3.31∗	2.52
HNR (dB)	20.92∗	7.60	23.77∗	6.76
SNR (dB)	21.19∗	7.09	23.87∗	6.59
NNE (dB)	−7.55∗	5.05	−8.45∗∗	4.94
STD F0 (Hz)	4.65∗∗	4.45	2.82∗∗	2.09
Mode F0 (Hz)	**264.34**	90.00	206.50∗∗	65.12
STD period (ms)	0.09∗∗	0.13	**0.08**	0.08
Mean amplitude (%)	82.91∗	8.34	86.43∗	6.28
STD amplitude (%)	7.97∗∗	3.94	6.53∗	2.72
F0 tremor (Hz)	**6.64**	3.67	**5.66**	3.51
Amplitude tremor (Hz)	**6.82**	3.68	**5.72**	3.58

Acoustic Values	AC			
Female		Male	
Mean	STD	Mean	STD
Jitter (%)	0.89∗	1.09	1.06∗∗	1.37
Shimmer (%)	4.25∗	3.14	6.80∗∗	6.35
HNR (dB)	20.86∗	6.43	18.88∗∗	8.86
SNR (dB)	21.13∗	6.17	19.32∗∗	8.25
NNE (dB)	−6.78∗	5.34	−6.11∗∗	5.80
STD F0 (Hz)	4.34∗	5.34	5.99∗∗	8.06
Mode F0 (Hz)	238.50∗	81.64	200.61∗	99.62
STD period (ms)	0.08∗∗	0.07	0.16∗	0.16
Mean amplitude (%)	**84.83**	7.36	80.10∗∗	9.30
STD amplitude (%)	7.15∗	3.87	9.38∗∗	4.49
F0 tremor (Hz)	**7.18**	4.01	**6.43**	3.67
Amplitude tremor (Hz)	**6.76**	3.69	7.13∗	4.16

*Notes:* ∗ Indicates a significant and ∗∗ a high significant difference between controls and the pathological group. The bold values exhibit no significant difference between controls and pathological voices. Comparisons are performed separately for both genders.

*Abbreviations:* Mean, mean values; STD, standard deviations; RLNP, paralysis of recurrent laryngeal nerve; FD, functional dysphonia; AC, anatomical changes; HNR, harmonic-to-noise ratio; SNR, signal-to-noise ratio; NNE, normalized noise error.

**Table 4:** Results of the Factor Analysis Separated for Men and Women

Acoustic Values	Female			Male		
1 (51.0%)	2 (13.7%)	3 (11.2%)	1 (56.8%)	2 (12.4%)	3 (10.6%)
Jitter (%)	**0.88**	0.07	0.01	**0.88**	0.10	0.12
Shimmer (%)	**0.92**	−0.02	0.05	**0.95**	0.05	0.01
HNR (dB)	**−0.90**	0.28	0.10	**−0.92**	0.21	0.18
SNR (dB)	**−0.89**	0.28	0.08	**−0.91**	0.21	0.20
NNE (dB)	**0.70**	−0.28	0.04	**0.79**	−0.14	−0.17
STD F0 (Hz)	**0.71**	0.51	0.13	**0.72**	0.43	0.41
Mode F0 (Hz)	−0.27	0.59	**0.64**	−0.21	**0.88**	0.29
STD period (ms)	**0.75**	0.09	−0.37	**0.79**	−0.18	0.26
Mean amplitude (%)	**−0.80**	−0.37	−0.14	**−0.84**	−0.03	−0.21
STD amplitude (%)	**0.74**	0.40	0.11	**0.83**	0.07	0.23
F0 tremor (Hz)	0.19	−0.39	**0.72**	0.33	0.51	**−0.60**
Amplitude tremor (Hz)	0.23	**−0.59**	0.45	0.39	0.34	**−0.61**

*Note:* The bold values show the highest factor loadings for each acoustic parameter.

*Abbreviations:* HNR, harmonic-to-noise ratio; SNR, signal-to-noise ratio; NNE, normalized noise error.
